# Targeting p27 tyrosine phosphorylation as a modality to inhibit CDK4 and CDK2 and cause cell cycle arrest in breast cancer cells

**DOI:** 10.18632/oncoscience.427

**Published:** 2018-06-25

**Authors:** Stacy W. Blain

**Affiliations:** Departments of Cell Biology and Pediatrics, SUNY Downstate Medical Center, Brooklyn, NY 11203, USA

**Keywords:** p27Kip1, cdk4, cdk2, palbociclib, breast cancer

The Cdk4/6 targeting drugs (CDK4i), Palbociclib, Abemaciclib, and Ribociclib, are approved in combination with Estrogen-targeting therapies, such as Letrozole or Fulvestrant, as frontline treatments for metastatic, hormone responsive (ER/PR+), Her2-patients and represent a unique class specifically approved for metastatic disease [1]. Cyclin D-cdk4/6, along with cyclin E-cdk2, controls the G1-S phase transition, and targeting these kinases has long been a type of holy grail in the oncology field. Cyclin D is downstream of most oncogenic signaling pathways, including the Estrogen and Her2 receptors, making cyclin D-cdk4/6 an attractive therapeutic target. However, while combined ER and cdk4/6 targeting significantly extends Progression Free Survival, patients develop resistance and Overall Survival is unchanged, suggesting that there is a need for identifying and specifically targeting these resistance mechanisms.

Cyclin D-cdk4/6′s main substrate is RB, which, when phosphorylated, allows the transcription of cyclin E [2]. Cyclin E-cdk2 complexes fully phosphorylate RB, and complete the G1-S phase transition. It has now been shown that in many cases increased activation of the downstream cdk2 kinase is responsible for resistance to CDK4i therapy. While others have shown that long-term exposure to CDK4i can cause amplification cyclin E [3], we have shown in ER+ breast cancer tissue culture models that resistance can occur within days, by epigenetic modulation of cdk2 activity [4]. In these resistant cells, cdk4/6 is still inhibited by Palbociclib, but now the cyclin-cdk regulator, p27Kip1, is degraded. This increases cdk2 activity, which compensates for the loss of cdk4/6 activity, and can phosphorylate RB and drive cells into S phase. This suggests that in order to provide durable arrest, inhibition at the onset of both the kinase that drives tumor progression (cdk4) as well as the kinase that will ultimately permit escape (cdk2) will be required.

**Figure 1:**
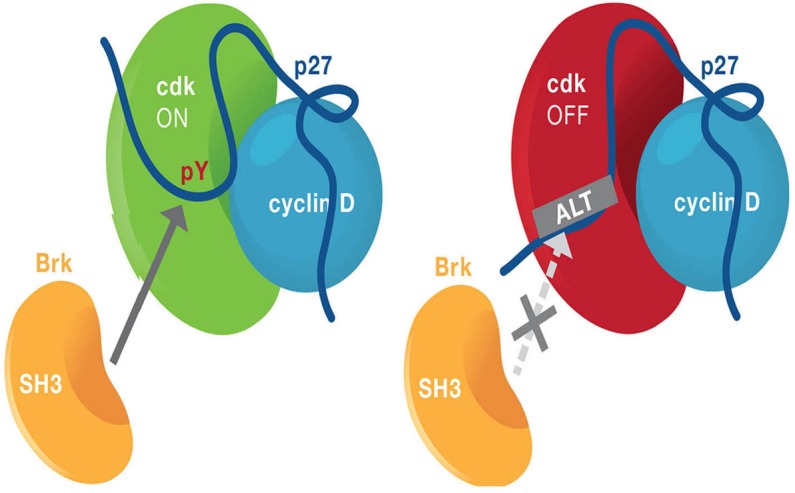
ALT blocks p27 Y88 phosphorylation and inhibits cdk4 and cdk2 activity Cartoon of the ALT:p27 interaction. Left: Brk interacts via its SH3 domain to bind p27 and then phosphorylate it on residue Y88, opening and activating the cdk4 complex. Right, ALT binds to p27, preventing Brk's interaction with p27 and blocking its phosphorylation on Y88, closing, and inactivating cdk4.

The cyclin D-cdk4 dimer is unstable and rapidly dissociates back into the inactive monomers, unless it associates with the p27 assembly factor. But, independent of its ability to assemble cyclin D-cdk4 complexes, p27 acts as a *bona fide* “switch” turning the complex on or off [5]. p27 associates with cdk4 in two alternative conformations: a closed, inactive or an open, activating conformation and the transition between these two forms is due to a conformational change following tyrosine (Y) phosphorylation on residue Y88 (or 89) in p27 itself (Figure 1). Breast tumor related kinase (Brk, so named because it is overexpressed in 60% of breast tumors) specifically phosphorylates Y88 to open and activate the complex [6]. This open complex now permits ATP access to the cdk4 active site, and also renders cdk4 competent to be phosphorylated on residue T172 by the Cyclin Activating Kinase (CAK), which fully activates kinase activity [5].

Y88 phosphorylation on p27 can therefore be thought of as a cdk4 ON/OFF switch [5]. However, p27's association with cdk2, whether Y phosphorylated or not, appears to always be inhibitory, due to p27's interaction with cyclin E and occlusion of the substrate binding site. However, *in vivo* Y88 phosphorylated p27 is a target for cdk2-dependent ubiquitin-mediated degradation, reducing p27's association with cdk2 and indirectly activating cyclin E-cdk2. This is logical: a cell that exits G0 wants to activate both cdk4 (p27 pY ON) AND cdk2 (increase p27-free cdk2), and rapid Y phosphorylation of p27, in response to mitogens, would achieve this. But, this would imply that the converse would also be true: preventing Y88 phosphorylation would inactivate cdk4 and cdk2. So if one wanted to generate a modality to inhibit both cdk4 (which drives tumors) and cdk2 (which would ultimately mediate resistance), targeting Y88 phosphorylation would achieve this.

Using a splice variant of Brk (ALT), which contains the SH3 domain, we demonstrate that its overexpression blocks Y88 phosphorylation, which then prevents p27 degradation, inhibits both cdk4 and cdk2 and arrests cells in G1, creating a more durable arrest than that seen with cdk4 inhibition alone (Figure 1) [4, 6]. When pY88 is inhibited, cdk1 remains active and cell viability is unchanged, consistent with p27's higher affinity for cdk2. When ALT was combined with Palbociclib, cells became senescent and were unable to reenter cycle after drug removal, which was different than Palbociclib-mediated arrest where cells reentered cycle upon drug removal. Finally, using a ER+ breast cancer cell derived xenograft model (MCF7), ALT expression or ALT + Palbociclib dual treatment resulted in tumor regression, whereas Palbociclib treatment just slowed tumor growth kinetics.

While this study helps to identify CDK4i resistance mechanisms, it also demonstrates that targeting pY88 might be a viable way of impacting two targets for the price of one. Cdk2 specific inhibitors have been difficult to generate due to the high degree of homology with the essential cdk1, and the toxic nature of these inhibitors resulted in numerous unsuccessful clinical trials. Targeting p27 might circumvent these issues. This study also suggests that phospho-p27 status serves as a surrogate marker for cdk4 activity, and may serve as a biomarker for patients to determine responsiveness to cdk4/6 inhibition. CDK4i are approved for metastatic ER+, Her2-breast cancer patients, but unlike other targeted therapies where clinical prescription is dependent on either proteomic or genomic verification of the target or target mutation, CDK4i are given to all metastatic patients in this subgroup. Cdk4 or cyclin D levels do not define cdk4 activity and in fact have been shown to not be clinical predictors of CDK4i response [2]. Identification of a CDK4i biomarker is an important area of research, especially as the CDK4i class of therapies hopes to expand into other clinical tumor indications.
